# Neuropathological changes associated with aberrant cerebrospinal fluid p-tau181 and Aβ42 in Alzheimer’s disease and other neurodegenerative diseases

**DOI:** 10.1186/s40478-024-01758-3

**Published:** 2024-03-27

**Authors:** Masanori Kurihara, Tomoyasu Matsubara, Satoru Morimoto, Akira Arakawa, Kensuke Ohse, Kazutomi Kanemaru, Atsushi Iwata, Shigeo Murayama, Yuko Saito

**Affiliations:** 1 Department of Neurology, Tokyo Metropolitan Institute for Geriatrics and Gerontology, Tokyo, Japan; 2 Department of Neuropathology (Brain Bank for Aging Research), Tokyo Metropolitan Institute for Geriatrics and Gerontology, Tokyo, Japan; 3 Integrated Research Initiative for Living Well with Dementia, Tokyo Metropolitan Institute for Geriatrics and Gerontology, Tokyo, Japan; 4 Department of Neurology, Tokushima University Hospital, Tokushima, Japan; 5 Department of Physiology, Keio University School of Medicine, Tokyo, Japan; 6 Brain Bank for Neurodevelopmental, Neurological and Psychiatric Disorders, United Graduate School of Child Development, Osaka University, Osaka, Japan

**Keywords:** Cerebrospinal fluid, Biomarkers, Tau proteins, Amyloid beta-peptides, Alzheimer's disease, Progressive supranuclear palsy, Corticobasal degeneration, Tauopathies, Dementia with lewy bodies, Neuropathology

## Abstract

Recent studies suggest that increased cerebrospinal fluid (CSF) phospho-tau is associated with brain amyloid pathology rather than the tau pathology. However, confirmation using gold standard neuropathological assessments remains limited. This study aimed to determine background pathologies associated with aberrant CSF p-tau181 and amyloid-beta 1–42 (Aβ42) in Alzheimer’s disease (AD) and other neurodegenerative diseases. We retrospectively studied all patients with antemortem CSF and postmortem neuropathologic data at our institution. Comprehensive neuropathologic assessments were conducted for all patients, including Thal phase, Braak NFT stage, and CERAD score for AD. CSF concentrations of p-tau181 and Aβ42 were compared between AD neuropathological scores at autopsy by one-way ANOVA stratified by other pathologies. A total of 127 patients with AD (*n* = 22), Lewy body disease (*n* = 26), primary tauopathies (*n* = 30), TDP-43 proteinopathy (*n* = 16), and other diseases (*n* = 33) were included. The age at lumbar puncture was 76.3 ± 9.1 years, 40.8% were female, and median time from lumbar puncture to autopsy was 637 (175–1625) days. While Braak NFT 0–II was prevalent without amyloid pathology, Braak NFT ≥IV was observed exclusively in patients with amyloid pathology. Stratified analyses showed that CSF p-tau181 was slightly but significantly higher in patients with high Thal phase or CERAD score even in those with Braak NFT 0–II at autopsy. In patients with amyloid pathology, CSF p-tau181 was significantly and more profoundly elevated in those with Braak NFT ≥III at autopsy. CSF Aβ42 was lower in patients with high amyloid pathological scores. However, 34% with Thal ≤ 2 and 38% with CERAD ≤ sparse also showed decreased Aβ42. Progressive supranuclear palsy (PSP) and corticobasal degeneration (CBD) were overrepresented in this group. These results neuropathologically confirmed previous studies that CSF p-tau181 levels were slightly elevated with amyloid pathology alone and were even higher with tau pathology, and that CSFAβ42 can be decreased in PSP/CBD.

**Supplementary Information:**

The online version contains supplementary material available at 10.1186/s40478-024-01758-3.

## Introduction

Alzheimer’s disease (AD) is neuropathologically characterized by the presence of extracellular amyloid-beta (Aβ) plaques and hyperphosphorylated tau in neurofibrillary tangles (NFT) in the brain. While varying degrees of amyloid plaques or transentorhinal NFT can be observed without symptoms, neocortical expansion of NFT is observed only in patients with a certain degree of amyloid pathology and is associated with brain atrophy and cognitive symptoms [1, 2].

In the CSF, decreased Aβ 1–42 (Aβ42) and increased phosphorylated tau (p-tau) are observed, and these biomarkers have been validated for the diagnosis of AD [3]. Changes in these biomarkers in relation to AD neuropathologic changes have been validated repeatedly [4–14]. The measurement of mid-region CSF tau phosphorylated at threonine 181 (p-tau181) is covered by insurance in Japan and in other countries and is widely conducted. While the current research framework assumes that these biomarker changes in Aβ42 and p-tau directly reflect the presence of Aβ or tau pathology, respectively [15], pathological evidence in this regard is insufficient and several questions remain.

First, the pathological background of increased CSF p-tau has been questioned in recent years. Longitudinal studies have shown that increased CSF p-tau is observed soon after Aβ abnormality and long before the detection of increased tau PET signals; therefore, it is now widely considered that CSF p-tau is more likely related to increased phosphorylation or secretion of tau from neurons associated with brain amyloid pathology [16–19]. However, many previous studies have shown that CSF p-tau is also associated with brain tau pathology and that previous human neuropathologic evidence was insufficient considering the possibility of confounding factors that may influence the known relationship between amyloid and tau pathology [20]. Second, although many previous studies have reported that CSF Aβ42 can also be decreased in patients with neurological diseases other than AD, such as dementia with Lewy bodies (DLB), progressive supranuclear palsy (PSP), and corticobasal degeneration (CBD) [9, 21–25], it is not known whether these decreases could be explained as being due to concomitant amyloid pathology. A hypothesis-driven analysis using a large autopsy-confirmed cohort with a balanced distribution of AD neuropathologic changes [20] may address these questions.

Therefore, this study aimed to investigate whether increased CSF p-tau is related to amyloid pathology, tau pathology, or both, and whether CSF Aβ42 can be decreased in the absence of amyloid pathology in certain disease pathologies.

## Methods

### Standard protocol approvals, enrollment, and patient consent

This retrospective study was approved by the Institutional Review Board of the Tokyo Metropolitan Institute for Geriatrics and Gerontology (approval number: R22-086). Written informed consent was obtained from the patients’ families before the autopsy. This study was performed in accordance with the tenets of the Declaration of Helsinki.

### Participants and setting

We retrospectively reviewed all patients who had undergone CSF biomarker testing at our institution from January 1995 to June 2023. Our institution is located in a suburban area of Tokyo, Japan, and provides community-based general and emergency services for the older adult population, including patients with dementia or neurodegenerative diseases. AD biomarkers were measured in all patients who had undergone a lumbar puncture, regardless of suspicion of clinical AD. Of the 4,832 patients who underwent CSF analysis during this period, all patients autopsied at our institution were included in this study. In addition to those who died at our hospital, patients who consented and registered for brain donation to the Brain Bank for Aging Research (BBAR) and subsequently died elsewhere were also autopsied at our institution. BBAR registrants include, but are not limited to, patients with AD.

### Biomarker measurements

CSF was obtained through a standard lumbar puncture. The first tube was sent for cell counting and routine biochemical testing, and subsequent CSF samples were collected directly into polypropylene tubes and stored at -30 °C until measured and at -80 °C for further storage. CSF concentrations of Aβ42 were measured using enzyme-linked immunosorbent assay (ELISA) (INNOTEST®, Fujirebio Europe N.V., Gent, Belgium). CSF concentrations of mid-region t-tau and p-tau181 were measured using ELISA (INNOTEST® or Finoscholar®, Nipro Corp., Osaka, Japan, an assay with identical characteristics distributed in Japan [26]) in accordance with the manufacturer’s protocol. The institutional cut-offs for p-tau181, 50.0 pg/mL; t-tau, 300 pg/mL; and Aβ42, 500 pg/mL, have previously been established [9, 27]. ApoE phenotyping was performed using isoelectric focusing, followed by Western blotting using serum or postmortem brain tissue.

### Neuropathologic evaluation

All patients, regardless of clinical diagnosis, underwent a thorough neuropathologic evaluation according to our BBAR protocol [28–31] in accordance with current guidelines. Briefly, the brain was divided in half, and one half was fixed in 20% buffered formalin for 1 to 2 weeks. The other half was frozen for future research, but representative portions were sampled before freezing and fixed in 4% paraformaldehyde for 48 h for diagnosis [28]. Representative anatomic areas were sampled, embedded in paraffin, and 6 μm-thick sections were used. Staining included Gallyas-Braak silver impregnation, and immunostaining was visualized with DAB using primary antibodies against human β-amyloid (12B2, dilution 1:50 with formic acid for antigen retrieval; IBL, Gunma, Japan), phosphorylated tau (AT8, dilution 1:1,000; Innogenetics, Ghent, Belgium), phosphorylated α-synuclein (pSyn#64, dilution 1:20,000 with formic acid for antigen retrieval; a gift from T. Iwatsubo, Japan; now available for purchase from FUJIFILM Wako Pure Chemical Corp, Osaka, Japan), and phosphorylated TAR DNA-binding protein 43 (TDP-43) (pSer409/410, dilution 1:10,000 with microwave in Dako target retrieval solution [pH 6.0] for antigen retrieval; a gift from M. Hasegawa, Japan; now available for purchase from Cosmo Bio, Tokyo, Japan).

Neuropathologic diagnoses and stagings were performed according to internationally accepted neuropathologic criteria for the diagnosis of AD [32, 33], Lewy body disease [31, 34, 35], PSP [36], CBD [37], frontotemporal lobar degeneration with TDP-43 proteinopathy [38], argyrophilic grain disease [30], and multiple system atrophy [39]. Those with spinocerebellar degeneration without α-synuclein pathology suggestive of multiple system atrophy were classified as spinocerebellar degeneration. Cerebrovascular disease was confirmed by macroscopic and microscopic evaluation. The diagnosis of Creutzfeldt-Jakob disease was based on Western blotting, genetic analysis, and neuropathology [40]. For AD neuropathology, Braak senile plaque and NFT stages [41, 42], the Consortium to Establish a Registry for Alzheimer’s Disease (CERAD) score [43], and Thal phase [44] were evaluated. Cerebral amyloid angiopathy staging was modified from a previous report [45] (0, none; 1, positivity confined to small vessel walls; 2, destruction of the small vessel wall and positivity extending to vascular smooth muscle; and 3, same as 2 plus resultant hemorrhage). The severity of Lewy body pathology was graded according to BBAR Lewy body stage as previously described [29, 31, 46]. For each patient, the pathology that most likely explained the clinical syndrome was used as the neuropathologic diagnosis to group patients. The control group was selected based on Thal phase 0–1, CERAD none, and no other significant pathological protein deposition (cerebrovascular changes were allowed except in the presence of documented dementia or parkinsonism).

### Statistical methods

Statistical analyses were conducted using GraphPad Prism version 9 (GraphPad Software, San Diego, CA, USA) or R version 4.0.3 (R Foundation for Statistical Computing, Vienna, Austria) and a graphical interface EZR (Saitama Medical Center, Jichi Medical University, Saitama, Japan). Missing data were treated using pairwise deletion. Categorical variables are expressed as percentages. Differences between groups were evaluated using Fisher’s exact test. Pairwise comparisons were performed using Holm’s method. Continuous variables with a normal distribution are presented as mean ± standard deviation, and continuous variables with a non-normal distribution are presented as medians (interquartile ranges). Differences between groups were evaluated using one-way analysis of variance (ANOVA), followed by *post hoc* analyses using a Dunnett test against the normal control or the lowest AD pathological score, or the Tukey test for analysis between multiple pathological diagnoses. Violin plots were colored with gradation based on q-values (the difference between means divided by the standard error of difference) only when one-way ANOVA was significant. Analysis for CSF p-tau181 was stratified according to amyloid and tau stages, respectively, to control for confounding. Pathological scores were grouped when necessary in reference to the ABC score in the NIA-AA guideline [32, 33] to ensure that each group had ≥ 4 patients. *P*-values < 0.05 were considered statistically significant for all analyses.

Multiple regression analysis was conducted for CSF p-tau181 with age, sex, the time interval from lumbar puncture to autopsy, *APOE* ε4 status (carrier or noncarrier), and postmortem AD pathology scores as independent variables. The performance of CSF p-tau181 to distinguish amyloid or tau pathology was assessed using receiver operating characteristic (ROC) analyses. Optimal cut-offs were determined by maximizing the Youden index.

## Results

### Baseline characteristics

Of the 4,832 patients who underwent CSF biomarker analysis during the study period, autopsy results were available for 141 patients. We included 127 patients with at least one AD CSF biomarker result. Group characteristics according to neuropathologic diagnosis are summarized in Table 1. Age at lumbar puncture was 76.2 ± 9.0 years and was similar between groups. The time interval from lumbar puncture to ELISA measurement was within 2 months. The time interval from lumbar puncture to autopsy was 623 (170–1,620) days, tending to be longer in the AD group and shorter in the TDP proteinopathy and ‘other’ groups. Aβ42, p-tau181, and t-tau results were available in 100%, 56.7%, and 99.2% of cases, respectively. Antemortem CSF Aβ42 levels were significantly lower, and p-tau181 and t-tau levels were significantly higher in patients with a neuropathologic diagnosis of AD than those with other diseases (Suppl. Figure 1).

**Table 1:** Group characteristics according to neuropathologic diagnosis

	AD	PD(D)/DLB	Primary tauopathy	TDP proteinopathy	Other	Multiple comparison p values	Total
n	22	26	30	16	33		127
Subtype (n)	AD 22	DLB 11, PDD 10, PD 5	PSP 18, CBD 4, PiD 1, DG/AGD 7	ALS ± FTD 12, FTLD-TDP 4	CVD 11, MSA 4 SCD 3, CJD 2 other 13		
Age at LP (years)	77.9 ± 7.9	77.9 ± 7.3	78.1 ± 6.3	76.3 ± 5.9	72.2 ± 12.6	0.045* (F = 2.5)	76.2 ± 9.0
LP to ELISA (days)	7 (2–20)	16 (9–27)	22 (8–54)	12 (7–55)	18 (9–41)	0.27 (F = 1.3)	17 (7–44)
LP to autopsy (days)	1107 (363–2538)	860 (177–1948)	556 (172–1277)	249 (183–446)	379 (80–1408)	0.66 (F = 0.6)	623 (170–1620)
Sex (female, %)	40.9%	42.3%	36.7%	62.5%	33.3%	0.41	40.9%
*APOE* ε4 carrier	59.1%	19.2%	20.7%	26.7%	12.9%	0.0047**	26.0%
ADNC							
none	0	4	6	4	11		25
low	0	18	18	11	18		65
intermediate	8	4	6	1	4		23
high	14	0	0	0	0		14
CSF biomarkers							
Aβ42 results	22/22 (100%)	26/26 (100%)	30/30 (100%)	16/16 (100%)	33/33 (100%)	1	127/127 (100%)
Aβ42 (pg/mL)	258 ± 111	605 ± 317	460 ± 262	537 ± 233	601 ± 324	< 0.001† (F = 6.7)	501 ± 294
p-tau181 results	14/22 (63.6%)	16/26 (62%)	18/30 (60%)	12/16 (75%)	12/33 (36.4%)	0.73	72/127 (56.7%)
p-tau181 (pg/mL)	79.4 ± 34.6	41.9 ± 12.1	38.9 ± 20.1	44.3 ± 14.7	39.2 ± 9.4	< 0.001† (F = 10.1)	48.4 ± 25.1
t-tau results	22/22 (100%)	26/26 (100%)	30/30 (100%)	16/16 (100%)	32/33 (97.0%)	1	126/127 (99.2%)
t-tau (pg/mL)	577 ± 354	146 ± 87	140 ± 118	231 ± 136	266 ± 255	< 0.001† (F = 16.4)	261 ± 261

*Abbreviations* Aβ42, amyloid-beta 1–42; AD, Alzheimer’s disease; ADNC, AD neuropathologic change; AGD, argyrophilic grain disease; ALS, amyotrophic lateral sclerosis; *APOE*, apolipoprotein E CBD; corticobasal degeneration; CJD, Creutzfeldt-Jakob disease; CSF, cerebrospinal fluid; CVD, cerebrovascular disease; DG, dementia with grains; DLB, dementia with Lewy bodies; ELISA, enzyme-linked immunosorbent assay; FTD, frontotemporal dementia; FTLD-TDP, frontotemporal lobe degeneration with TDP-43 pathology; LP, lumbar puncture; MSA, multiple system atrophy; PD(D), Parkinson’s disease (with dementia); PiD, Pick’s disease; PSP, progressive supranuclear palsy; p-tau181, tau phosphorylated at threonine 181; SCD, spinocerebellar degeneration; TDP-43, TAR DNA-binding protein 43; t-tau, total tau. * Post-hoc Tukey test showed no significant difference between groups. ** Pairwise comparison showed a significantly higher frequency in AD compared to Other group, and a higher trend in AD compared to Primary tauopathy group (*p* = 0.065) and PD(D)/DLB group (*p* = 0.063). † Plots and results of post-hoc comparisons are shown in Supplementary Fig. 1

The distributions of AD neuropathologic changes are summarized in Suppl. Figure 2. While Braak NFT stages I and II (transentorhinal stages) were frequently observed and stage III was occasionally observed with no or low amyloid pathology in this older adult cohort, Braak NFT stages IV, V, and VI were exclusively observed with advanced amyloid pathology.

### CSF biomarkers according to AD neuropathologic scores

**Fig. 1:**
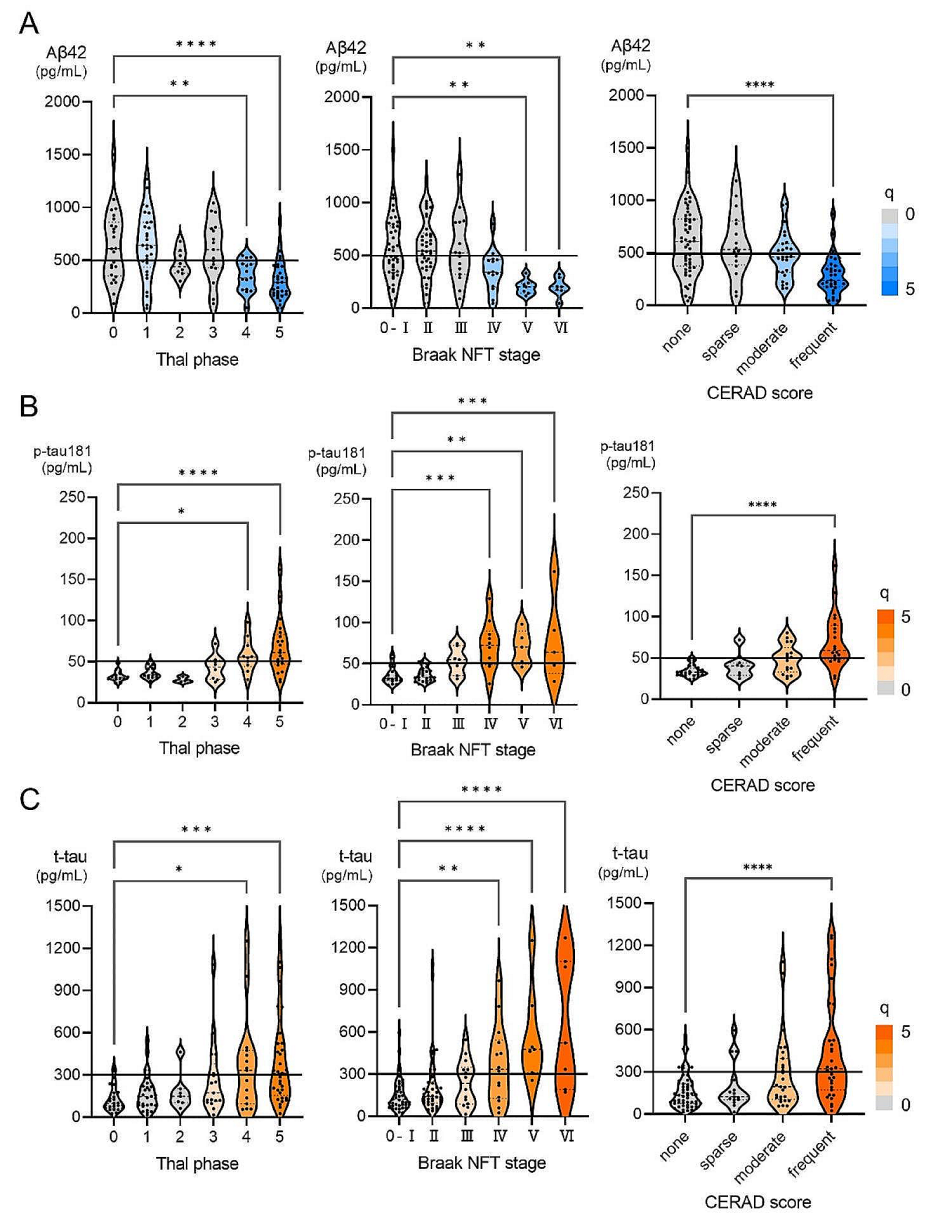
CSF AD Biomarkers according to AD Neuropathologic Scores. (**A**) CSF Aβ42 levels compared between patients grouped by AD neuropathologic scores at autopsy. (**B**) CSF p-tau181 levels compared between patients grouped by AD neuropathologic scores at autopsy. (**C**) CSF t-tau levels compared between patients grouped by AD neuropathologic scores at autopsy. *Note* Horizontal lines represent pre-determined cut-off values

CSF Aβ42, p-tau181, and t-tau levels were compared between patients grouped according to postmortem AD neuropathologic changes, Thal phase, Braak NFT stage, and CERAD score. CSF Aβ42 was lower in patients with advanced pathology in all AD neuropathologic scores, Thal phases, Braak NFT stages, and CERAD scores (Fig. 1A). CSF p-tau181 and t-tau levels were higher in patients with advanced pathology in all AD neuropathologic scores, Thal phases, Braak NFT stages, and CERAD scores (Fig. 1B, C).

### Associations between CSF tau and amyloid or tau pathology stratified according to other pathology

**Fig. 2:**
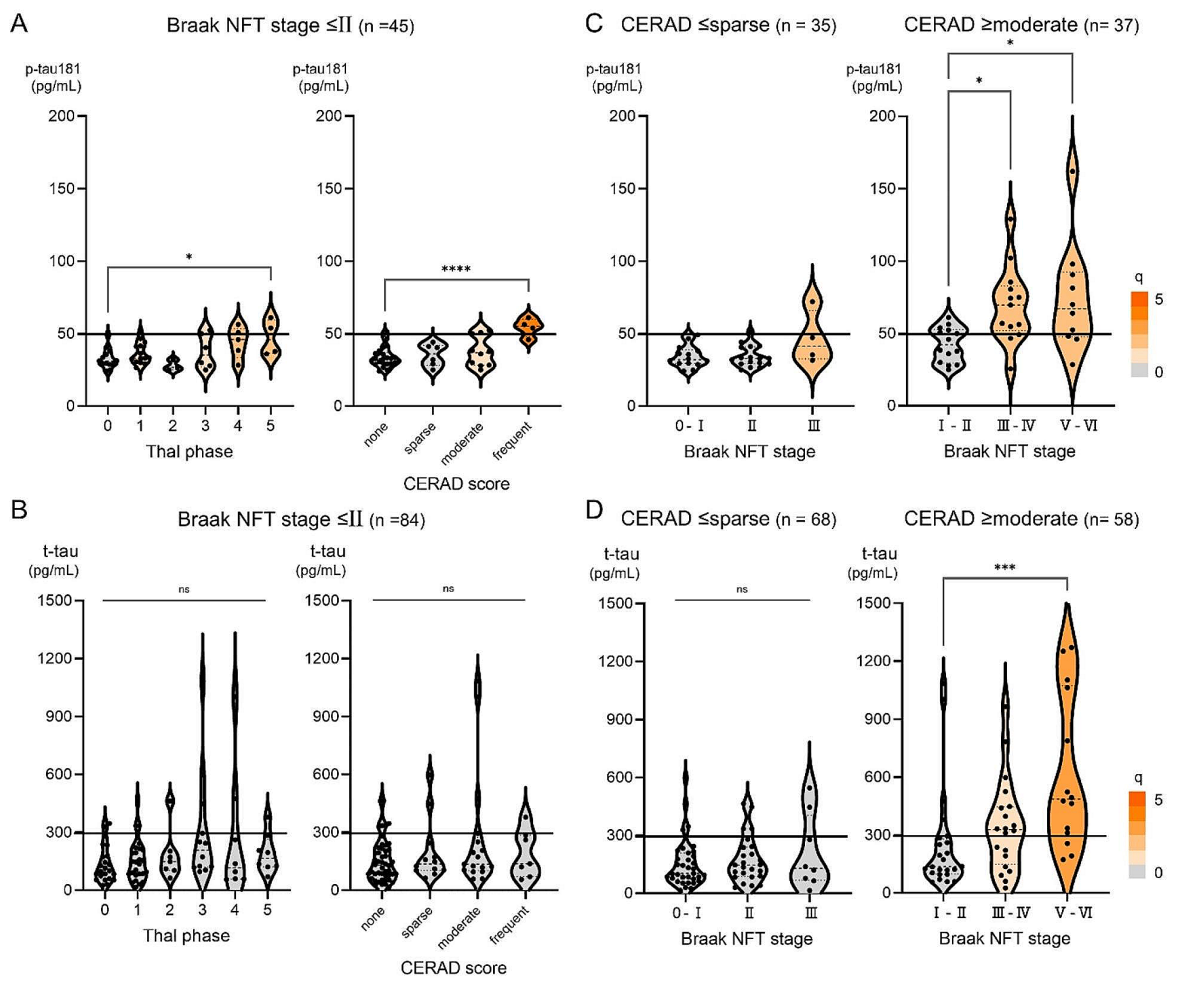
CSF p-tau181 and t-tau according to AD Neuropathologic Scores Stratified according to other Pathology. (**A**) In a subgroup of patients with a low Braak NFT stage (≤II) at autopsy, antemortem CSF p-tau181 was slightly but significantly higher in patients with high amyloid pathologic scores compared with those with no amyloid pathology. (**B**) The difference in CSF t-tau between patients grouped by amyloid pathologic scores in a subgroup of patients with a low Braak NFT stage (≤II) was not significant. (**C**) Stratified according to the severity of amyloid pathology, CSF p-tau181 tended to be higher with a high Braak NFT stage (III) in patients with a low CERAD score and was significantly higher with a high Braak NFT stage (≥III) in patients with a high CERAD score. (**D**) In patients with a high CERAD score, CSF t-tau was significantly higher with high Braak NFT stages (≥V) at autopsy in patients only among those with a high CERAD score

**Fig. 3:**
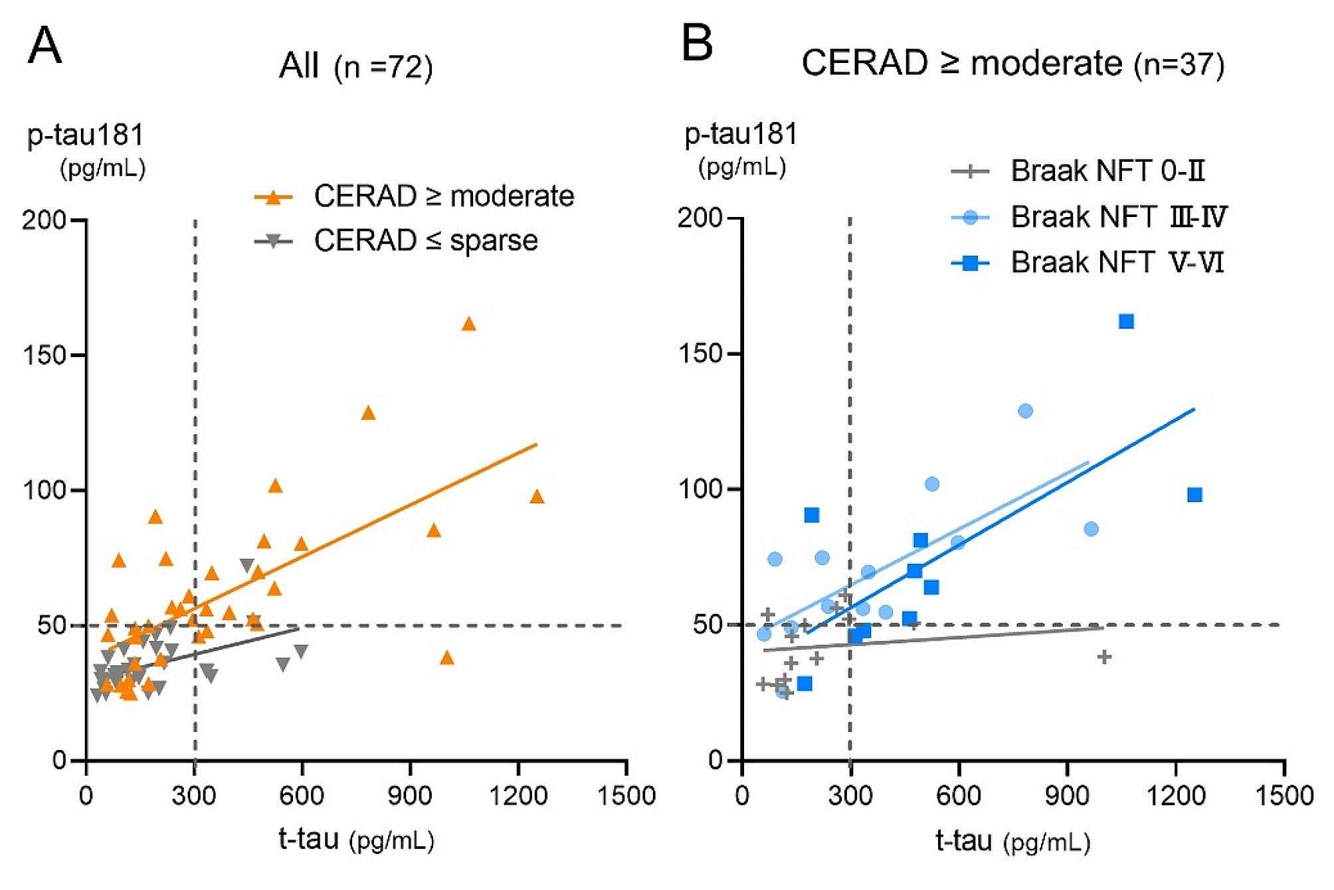
The Association between CSF p-tau181 and t-tau Stratified according to AD neuropathologic scores. (**A**) The distribution plots and Deming regression line showed different trends between CERAD scores lower than or equal to sparse and CERAD higher than or equal to moderate, suggesting a higher CSF p-tau181 in CERAD higher than or equal to moderate. (**B**) In a subgroup of patients with CERAD higher than or equal to moderate, the distribution plots and regression line showed different trends between Braak NFT stage ≤II and Braak NFT stage ≥III suggesting a higher CSF p-tau181 in Braak NFT stages ≥III

Given the known strong relationship between amyloid and tau pathology, which was also confirmed in our cohort (Suppl. Figure 2), we conducted stratified analyses for p-tau181 to control for confounding between amyloid and tau pathology. Even in the subgroup of patients with a low Braak NFT stage (0–II) at autopsy, CSF p-tau181 was slightly but significantly higher in patients with high amyloid pathology scores compared with those without amyloid pathology (Fig. 2A). The difference in CSF t-tau showed no significance in the same analysis (Fig. 2B). When stratified according to the postmortem CERAD score, CSF p-tau181 tended to be higher with a high Braak NFT stage (III) in patients with a low CERAD score (none or sparse), and was significantly higher with high Braak NFT stages (≥III) in patients with high CERAD scores (moderate or frequent) (Fig. 2C). The difference in CSF t-tau was significant only in those with a high CERAD score and was significantly higher in Braak NFT stages ≥V (Fig. 2D). The distribution plots and regression line between p-tau181 and t-tau showed different trends related to postmortem brain pathology, suggesting higher CSF p-tau181 levels in moderate or frequent CERAD scores and Braak NFT stage ≥III (Fig. 3).

Based on these findings, a multiple regression analysis was conducted with CSF p-tau181 as the dependent variable and age, sex, time interval from lumbar puncture to autopsy, *APOE* ε4 status (carrier or noncarrier), CERAD none-sparse or moderate-frequent, and Braak NFT stage ≤II or ≥III as independent variables. CSF p-tau181 levels were log-transformed for normal distribution. CERAD none-sparse or moderate-frequent (*t* = 2.79, *P* < 0.01) and Braak NFT stage ≤II or ≥ III (*t* = 4.71, *P* < 0.001) both remained significantly associated with CSF p-tau181 levels and were the only variables that remained significant.

ROC analyses using the entire sample showed that CSF p-tau181 had an area under the curve (AUC) of 0.81 (95% confidence interval [CI] 0.70–0.92) for predicting CERAD moderate-frequent and an AUC of 0.86 (95% CI 0.75–0.96) for predicting Braak NFT ≥III at autopsy, both at similar cut-off levels of 45.9 and 46.0 pg/mL, respectively. Limiting the analysis to Braak NFT stage ≤II to predict CERAD moderate-frequent showed a similar optimal cut-off of 45.9 pg/mL (AUC 0.69, 95% CI 0.50–0.89), while limiting the analysis to CERAD moderate-frequent to predict Braak NFT stage ≥III showed a higher cut-off of 63.9 pg/mL with 100% specificity (AUC 0.83, 95% CI 0.69–0.96).

### Characteristics of decreased CSF Aβ42 in patients without postmortem amyloid Pathology

**Fig. 4:**
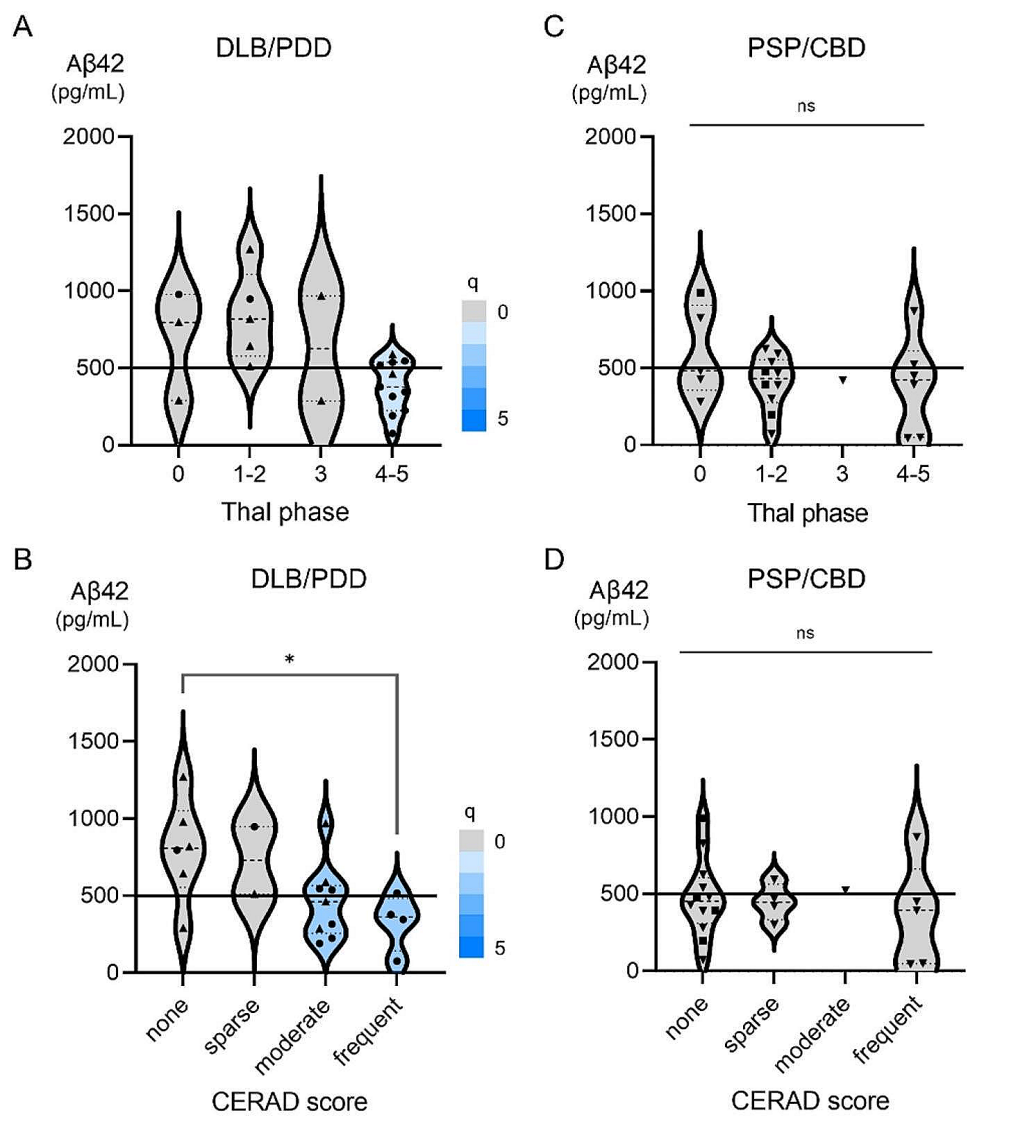
The association between CSF Aβ42 and Postmortem Amyloid Pathologic Scores in DLB/PDD and PSP/CBD. The study was performed in 21 patients with DLB/PDD (DLB, *n* = 11; PDD, *n* = 10) and 22 patients with PSP/CBD (PSP, *n* = 18; CBD *n* = 4). (**A**) A significant difference in CSF Aβ42 was observed between patients grouped according to postmortem Thal phase in DLB/PDD and tended to be lower in the highest Thal phase. (**B**) CSF Aβ42 was significantly lower in patients with frequent neuritic plaques in terms of the CERAD score at autopsy in DLB/PDD. (**C**, **D**) The difference in CSF Aβ42 was nonsignificant between patients grouped according to postmortem Thal phase or CERAD score in PSP/CBD. Decreased CSF Aβ42 below the cut-off was frequently observed in patients with a low Thal phase and CERAD score at autopsy. *Note* Circles, upward triangles, downward triangles, and squares represent data from patients with DLB, PDD, PSP, and CBD, respectively

While CSF Aβ42 was significantly lower in patients with high amyloid pathology scores, a significant number of patients with low amyloid pathology scores also had CSF Aβ42 below the cut-off (Fig. 1A; Suppl. Figure 3). Notably, 44% with Thal phase 0 and 42% with no neuritic plaques on CERAD had CSF Aβ42 levels below the cut-off (Suppl. Figure 3). None had amyloid angiopathy stage ≥ 2 on a scale of 0–3. Although various neuropathological diagnoses were observed in patients with decreased CSF Aβ42 levels and low amyloid pathology scores at autopsy, we found that primary tauopathy was overrepresented in our cohort, consisting mainly of PSP and CBD (Suppl. Figure 4). Compared with age-matched controls without significant brain pathology at autopsy, CSF Aβ42 was significantly lower in patients with a pathological diagnosis of PSP/CBD and tended to be lower in those with DLB/Parkinson’s disease with dementia (PDD) (Suppl. Figure 5). CSF Aβ42 was not associated with the severity of Lewy pathology (Suppl. Figure 6). While decreased CSF Aβ42 was associated with amyloid pathology scores in DLB/PDD (Fig. 4A, B), decreased CSF Aβ42 was not associated with amyloid pathology scores in PSP/CBD (Fig. 4C, D).

Antemortem measurements of both Aβ42 and Aβ40 via the currently used Lumipulse assay using the LUMIPULSE system (FUJIREBIO INC., Tokyo, Japan) were available for a single patient. While CSF Aβ42 was decreased below the cut-off, CSF Aβ40 was also low and the Aβ42/Aβ40 ratio was within the normal range [47] (Suppl. Figure 7). Neuropathologically, amyloid pathology was limited (Thal phase 2, CERAD none) and the neuropathological diagnosis was CBD (Suppl. Figure 7).

## Discussion

In this study, we confirmed neuropathologically that CSF p-tau181 levels were associated with both amyloid and tau pathology, even after stratification according to other pathology. While CSF Aβ42 was decreased in association with increasing amyloid pathology, a significant number of patients without amyloid pathology also had decreased CSF Aβ42.

CSF p-tau181 was increased in association with amyloid pathology even in the absence of tau pathology expansion. It has been well established that CSF p-tau181 is elevated in patients with AD [3]. While it seems natural to assume that elevated CSF p-tau181 is associated with tau pathology in the brain, recent evidence suggests that elevated CSF p-tau181 is more likely associated with increased phosphorylation or secretion of tau from neurons associated with amyloid pathology [16–19]. To further neuropathologically confirm this current notion in human patients, we compared CSF p-tau181 levels between patients grouped in relation to amyloid-related pathological scores stratified according to tau pathology in a relatively large cohort with various degrees of AD neuropathologic changes. Our results clearly showed that CSF p-tau181 was mildly but significantly elevated in patients with high amyloid pathological scores, even in those with limited tau pathology to the transentorhinal region (Braak NFT stage 0–II) at autopsy.

Moreover, CSF p-tau181 was even higher in patients with amyloid pathology plus Braak NFT ≥III. The correlation between CSF p-tau181 and t-tau in patients with high amyloid and tau pathology scores also suggested that CSF p-tau181 was not only an amyloid marker but also related to tau pathology ± neurodegeneration. A recent study investigating the relationship between plasma p-tau species and AD neuropathologic changes also suggested that p-tau181 levels are associated with both amyloid and tau pathology [48]. A recent study using a highly sensitive tau PET tracer, namely, ^18^F-MK6240, also showed a large CSF p-tau181 increase from PET-based Braak stage ≥III [49]. Our results and recent studies suggest that CSF p-tau181 is associated not only with amyloid but also with tau pathology. In other words, considering the chronological relationship between amyloid and tau pathology, CSF p-tau181 starts to be mildly elevated with the progression of amyloid pathology and continues to be more elevated with the expansion of tau pathology.

A revised NIA-AA clinical criteria for AD in draft proposes to classify tau biomarkers into several categories, including none, medial temporal lobe, and neocortical, rather than the current dichotomy of positive vs. negative. This categorization should be important for estimating the effect of AD pathology on clinical symptoms. The degree and extent of tau pathology may also be important to estimate the response to disease-modifying therapies [50]. However, these classifications are based on tau PET findings, and fluid p-tau is listed only as an early-stage amyloid biomarker based on binary interpretation. Increased t-tau may indicate neocortical expansion of tau pathology, although caution should be exercised because t-tau can be increased in several other conditions, including stroke, head trauma, or Creutzfeldt-Jakob disease [51]. Our study results suggest that the establishment of two or more cut-offs for CSF p-tau may be straightforward and have the potential to differentiate between patients into three groups; amyloid-negative, amyloid-positive but tau pathology limited in the transentorhinal region (Braak NFT 0–II), and amyloid-positive plus extensive tau pathology (Braak NFT ≥III).

While the predefined single cut-off for the diagnosis of AD was 50.0 pg/mL, based on our stratified ROC analyses, the cut-off was 45.9 pg/dL for differentiating amyloid-negative from amyloid-positive individuals and 63.9 pg/mL for differentiating tau pathology limited to the transentorhinal region from extensive tau pathology in amyloid-positive individuals. These values should differ between measurement platforms and need to be directly confirmed in the currently used fully automated immunoassays, but could be close to 54.3 and 83.1 pg/mL for the Lumipulse assay, and 16.3 and 24.2 pg/mL for the Elecsys assays based on previous Passing–Bablok regression lines [52, 53].

While CSF Aβ42 was lower in patients with high amyloid pathology scores at autopsy, as previously reported [5, 6, 8, 11, 12], a substantial proportion of patients with decreased CSF Aβ42 had low amyloid pathology scores at autopsy. Although the neuropathologic diagnosis varied widely, patients with PSP and CBD were overrepresented in our cohort among those with decreased CSF Aβ42 and low amyloid pathology scores at autopsy. While decreased CSF Aβ42 in patients with DLB/PDD [9, 22] was associated with concomitant amyloid pathology, known as the common form [54] as previously reported [21], decreased CSF Aβ42 in patients with PSP/CBD [23, 24] was not associated with amyloid pathology. It has been reported that decreased CSF Aβ42 in PSP may be associated with disease severity, which may lead to a decrease in total APP-derived peptides including both Aβ42 and Aβ40 [24]; therefore, using the current standard approach of using the CSF Aβ42/40 ratio [55] (as in the patient in Supplementary Fig. 7) may be more specific to evaluate brain amyloid pathology in PSP/CBD, as has been recently suggested [56].

This study had some limitations. First, although the number of patients was relatively large for a study directly comparing antemortem CSF with postmortem pathology, the sample size was not sufficiently large to control for other confounders. Second, the time interval between lumbar puncture and autopsy is always a limitation in this type of study. However, because the pathology was likely less advanced at the time of lumbar puncture, we consider the main conclusion of the study that CSF p-tau181 can be increased without significant tau pathology being sustained. Third, although we employed widely used standard pathological scores, differences within the same scores may have had additional effects. Additional analyses without ceiling effects, such as quantitative measurement of different forms of amyloid plaques, may be useful to test this hypothesis. Fourth, we used the results of manual ELISA, which may have increased the technical variation in the results compared with current automated methods. Fifth, we only obtained measurement results of CSF mid-region p-tau181 for p-tau, whereas the relationship between p-tau and amyloid or tau is known to differ between p-tau species [16, 19, 57].

## Conclusion

We neuropathologically confirmed that CSF p-tau181 was associated with both amyloid and tau pathology. CSF p-tau181 levels were slightly elevated with amyloid pathology alone and even higher with expansion of tau pathology. Decreased Aβ42 was observed in the absence of amyloid pathology in patients with neurological diseases such as PSP/CBD. These findings contribute to a better understanding of CSF AD biomarkers in relation to neuropathologic changes.

### Electronic supplementary material

Below is the link to the electronic supplementary material.

Supplementary Material 1

## Data Availability

The datasets used and/or analysed during the current study available from the corresponding author on reasonable request.

## Abbreviations

AD: Alzheimer’s disease
ANOVA: analysis of variance
Aβ: amyloid-beta
Aβ42: amyloid-beta 1–42
BBAR: Brain Bank for Aging Research
CBD: corticobasal degeneration
CERAD: Consortium to Establish a Registry for Alzheimer’s Disease
DLB: dementia with Lewy bodies
ELISA: enzyme-linked immunosorbent assay
NFT: neurofibrillary tangles
PDD: Parkinson’s disease with dementia
PSP: progressive supranuclear palsy
p-tau: phosphorylated tau
p-tau181: tau phosphorylated at threonine 181
ROC: receiver operating characteristic
TDP-43: TAR DNA-binding protein 43
