# Supplementary material for: Neuropathological changes associated with aberrant cerebrospinal fluid p-tau181 and Aβ42 in Alzheimer’s disease and other neurodegenerative diseases
Source: Acta Neuropathol Commun. 2024 Mar 27;12:48. doi: 10.1186/s40478-024-01758-3 (PMC10976730; doi:10.1186/s40478-024-01758-3)
Supplement: Supplementary file 1 — Supplementary Material 1 [file 40478_2024_1758_MOESM1_ESM.docx]

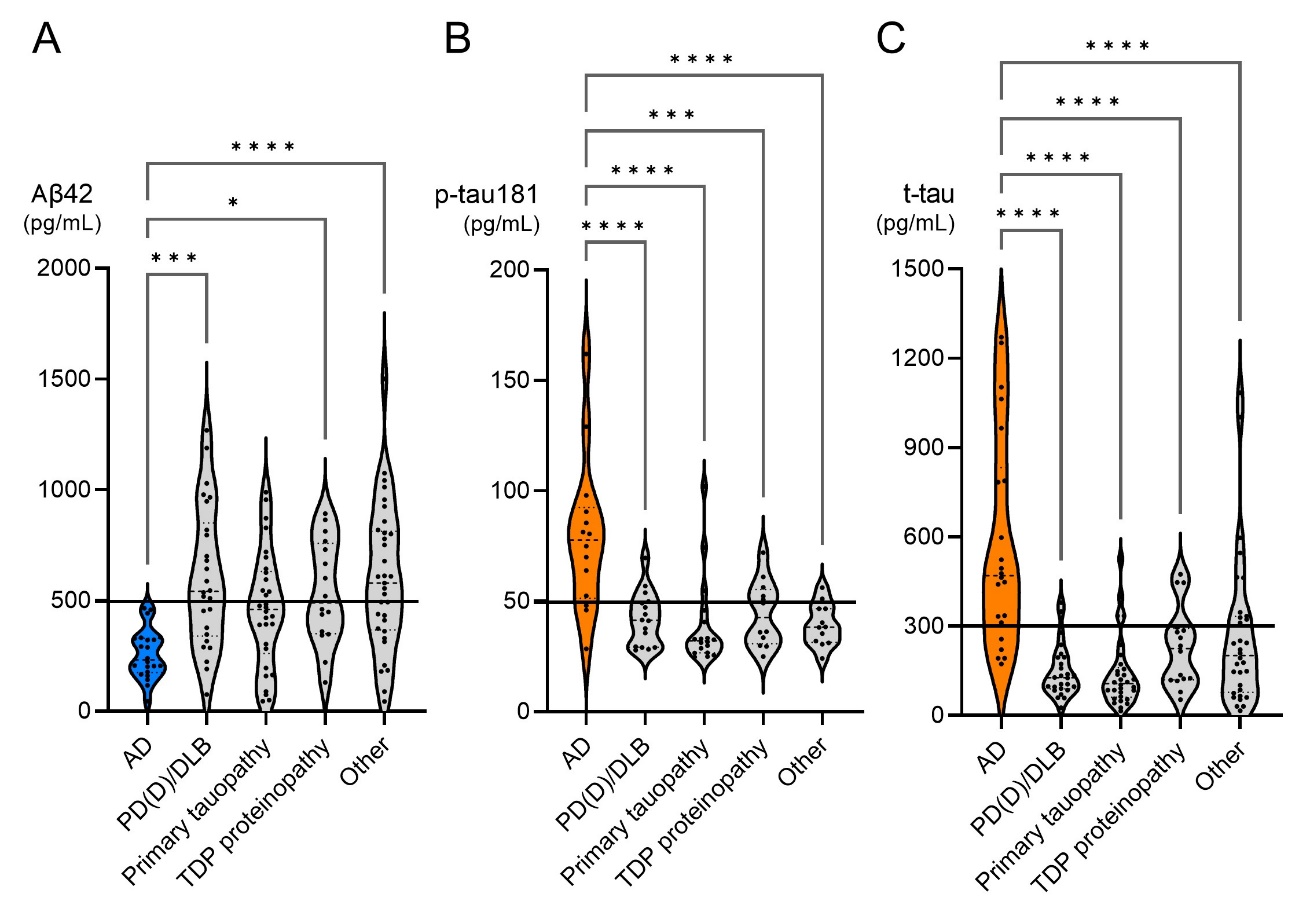


**Supplementary Figure 1. Cerebrospinal fluid (CSF) Alzheimer’s disease (AD) Biomarkers by Neuropathologic Diagnoses**

CSF Aβ42, p-tau181, and t-tau were significantly altered in patients with a postmortem diagnosis of AD compared to other diseases.


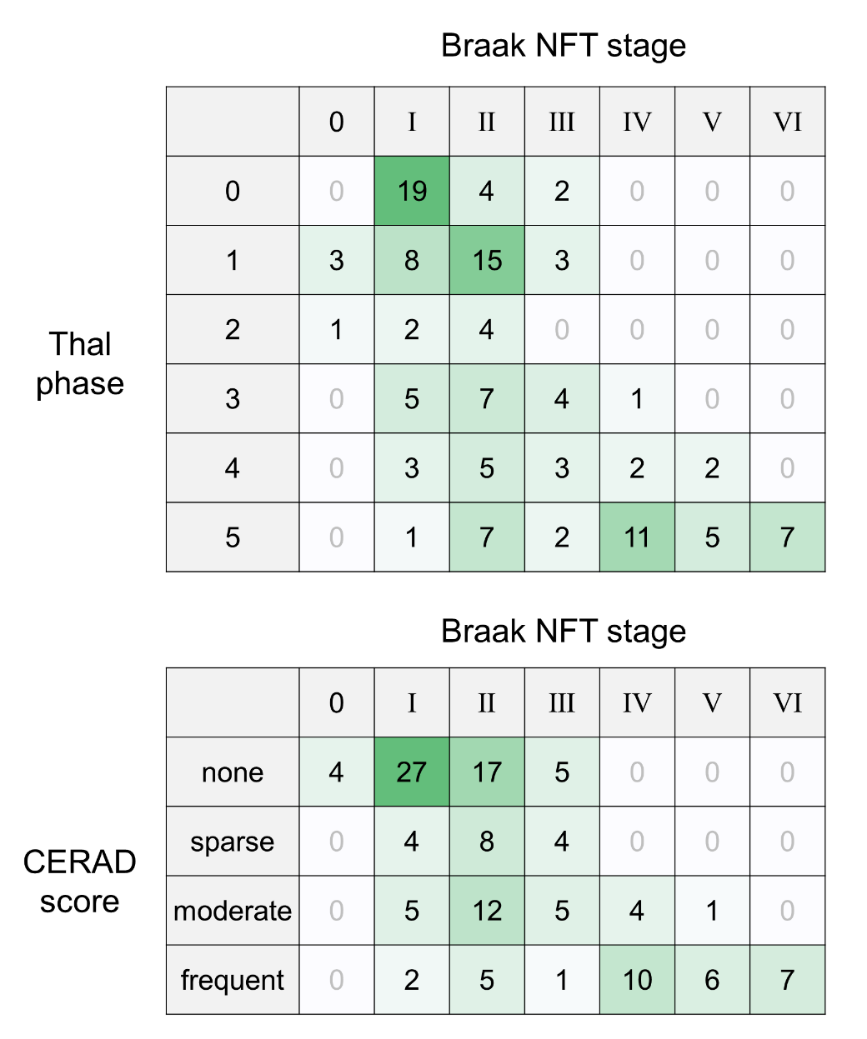


**Supplementary Figure 2. Heatmap Distribution of AD Neuropathologic Changes**

While Braak NFT stages Ⅰ and Ⅱ (transentorhinal stages) were commonly observed and stage Ⅲ was occasionally observed with no or low amyloid pathology in this older adult cohort, Braak NFT stages Ⅳ, Ⅴ, and Ⅵ were exclusively observed with advanced amyloid pathology.

Abbreviations: AD, Alzheimer’s disease; CERAD, Consortium to Establish a Registry for Alzheimer’s Disease; NFT, neurofibrillary tangle


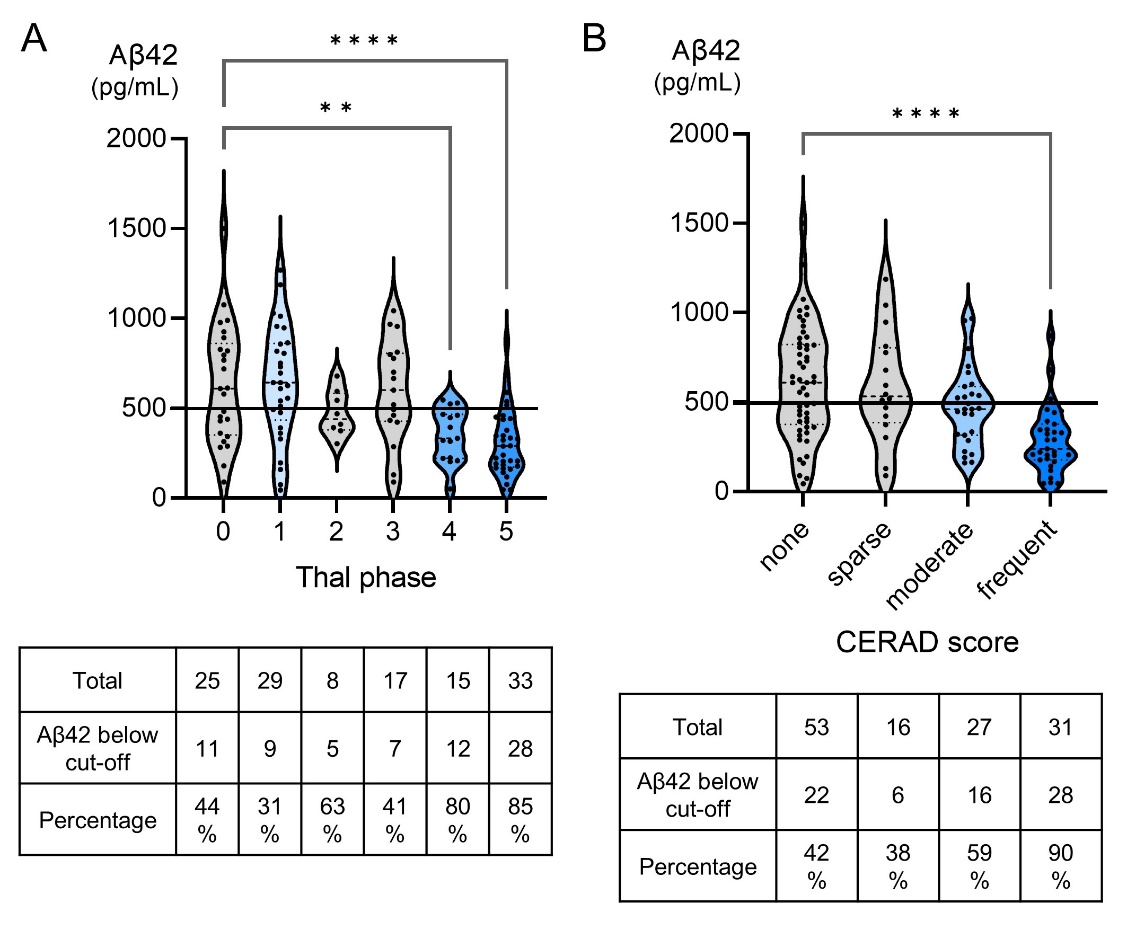


**Supplementary Figure 3. The Proportion of Decreased CSF Aβ42 Grouped by Postmortem Amyloid Pathologic Scores**

While the percentages of patients with decreased CSF Aβ42 were also higher in high postmortem Thal phase (A) and CERAD score (B), a substantial proportion of patients with low amyloid pathologic scores also had decreased CSF Aβ42 below the cut-off including 44% of patients with Thal phase 0 (A) and 42% with ”none” neuritic plaques on CERAD (B).


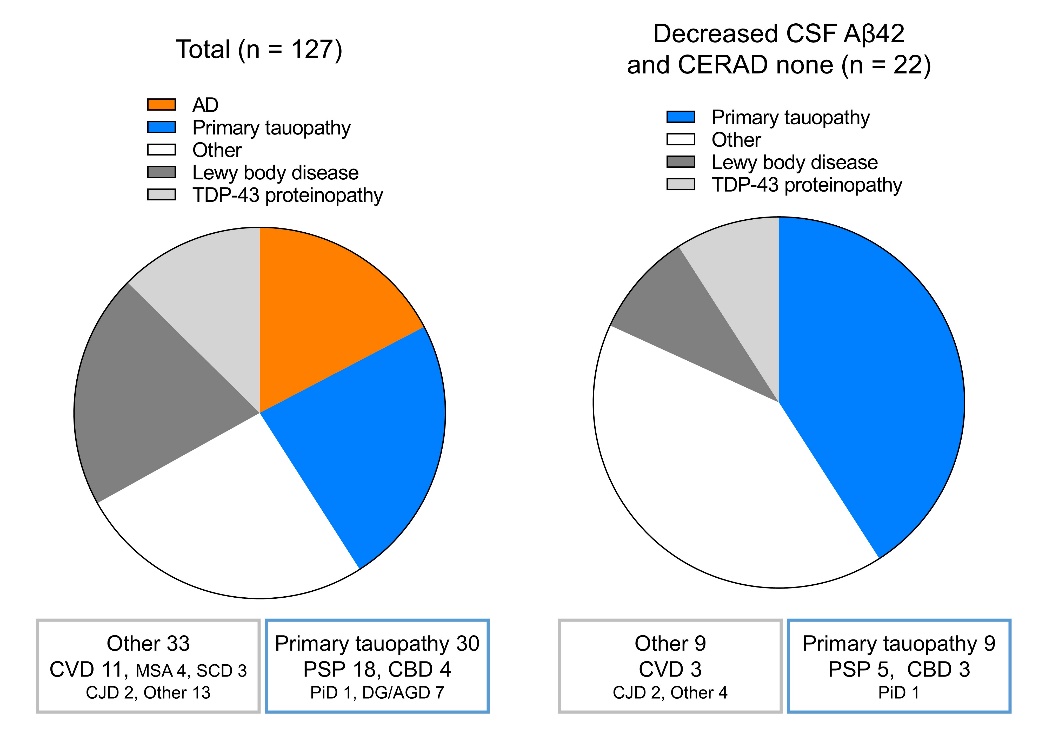


**Supplementary Figure 4. Proportion of Pathological Diagnoses in Patients with Decreased Aβ42 and “None” Neuritic Plaque on CERAD Score**

While patients with a pathological diagnosis of primary tauopathy represented 23.6% of the total cohort, primary tauopathy accounted for 40.9% and was the most common diagnostic group with decreased CSF Aβ42 and no neuritic plaques on CERAD at autopsy. Five patients with PSP and 3 patients with CBD were included. Diagnosies in patients with “Other” included meningitis, cerebritis, and intravascular lymphoma.

AD: Alzheimer’s disease, PSP: progressive supranuclear palsy, CBD: corticobasal degeneration, PiD: Pick's disease, DG: dementia with grains, AGD: argyrophilic grain disease, CVD: cerebrovascular disease, MSA: multiple system atrophy, SCD: spinocerebellar degeneration, CJD: Creutzfeldt-Jakob disease


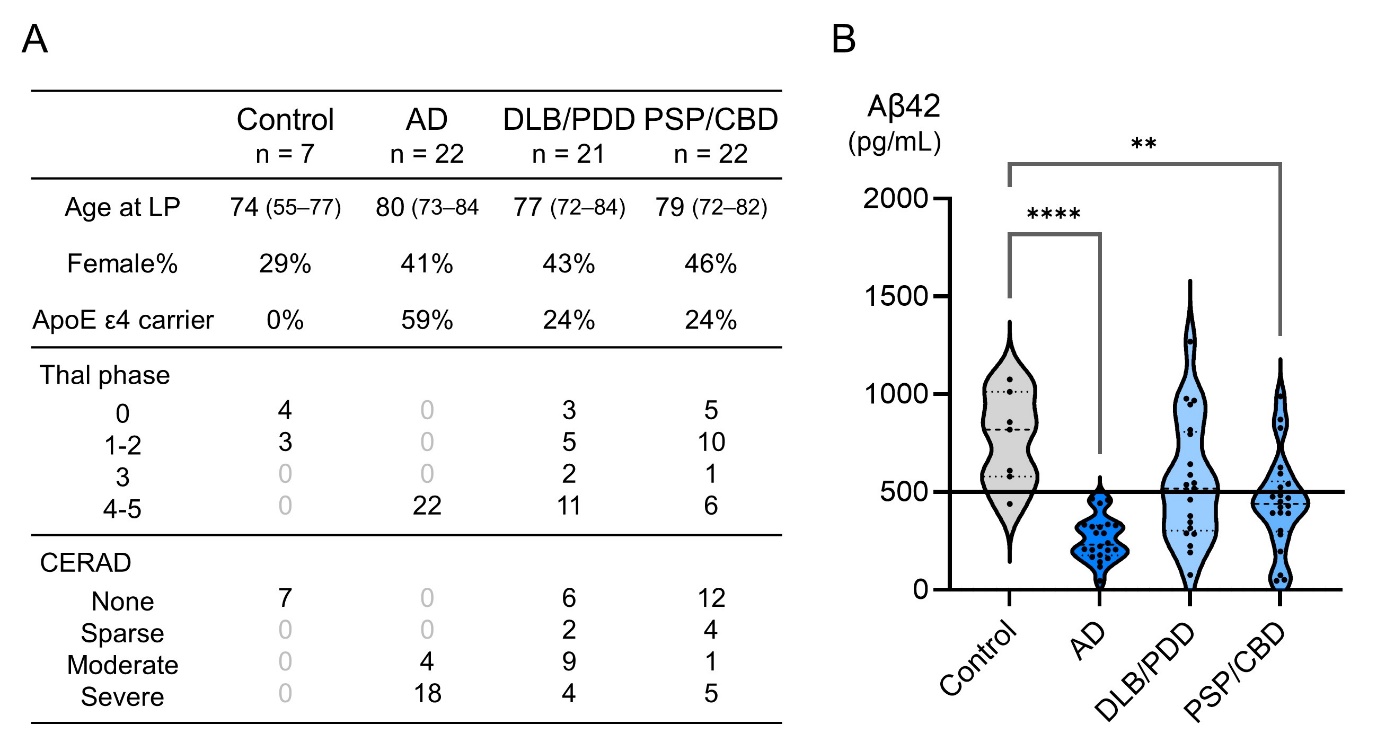


**Supplementary Figure 5. CSF Aβ42 in Control, AD, DLB/PDD, and PSP/CBD**

(A) Baseline characteristics of patients with AD, DLB/PDD, and PSP/CBD and age-matched controls without significant brain pathology. (B) CSF Aβ42 was significantly lower in AD and PSP/CBD and tended to be lower in DLB/PDD compared to control. Nine patients (42.9%) with DLB/PDD and 15 (68.2%) patients with PSP/CBD had CSF Aβ42 values below the cut-off.

AD: Alzheimer’s disease, DLB: dementia with Lewy bodies, PDD: Parkinson’s disease with dementia, PSP: progressive supranuclear palsy, CBD: corticobasal degeneration


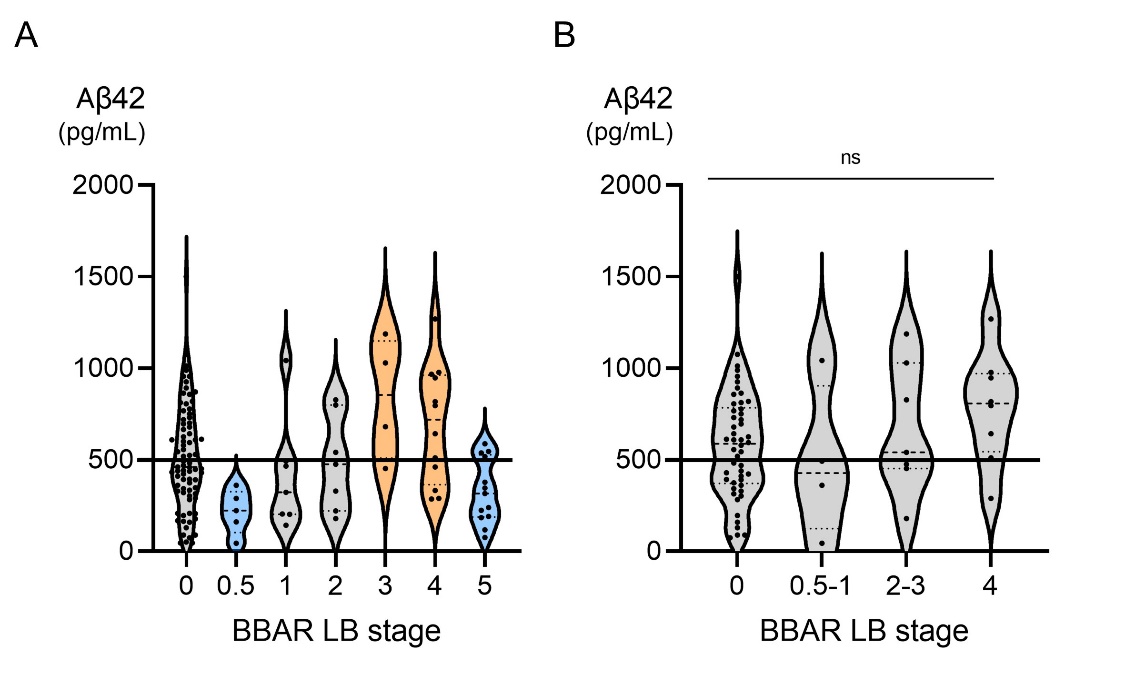


**Supplementary Figure 6. CSF Aβ42 by BBAR Lewy body stage**

CSF Aβ42 levels compared between patients grouped by Brain Bank for Aging Research (BBAR) Lewy body (LB) stage at autopsy. (A) Analysis of all patients (n = 127). Although one-way ANOVA was significant, post-hoc comparison showed no significant difference between groups and the trend was not consistent with increasing LB pathology. (B) Analysis limited to patients with none or sparce CERAD (n = 69). No difference between groups was observed.


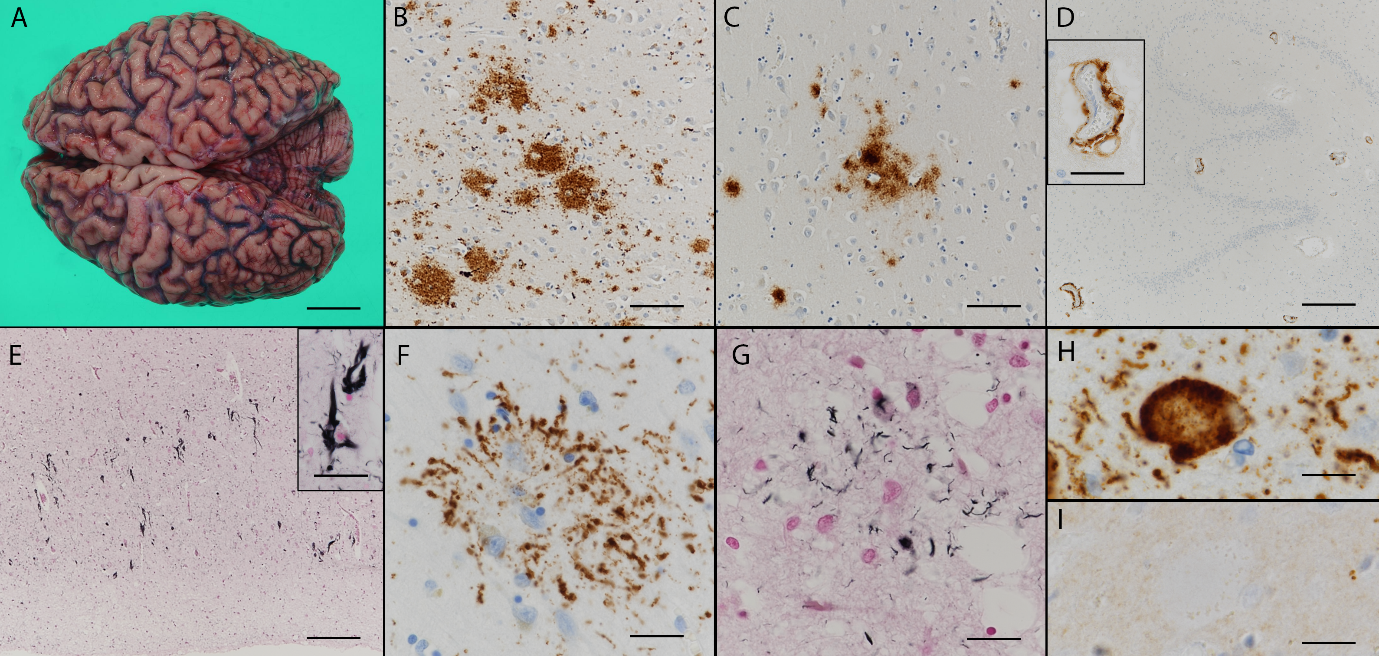


**Supplementary Figure 7. Representative findings in a patient with CBD**

A 76-year-old woman presented with a 4-year history of frontal cognitive decline and parkinsonism. CSF Aβ42 measured by Innotest ELISA was 393 pg/mL, which was below the cut-off (500 pg/mL). However, CSF Aβ42/Aβ40 ratio measured by Lumipulse assays using the LUMIPULSE system (FUJIREBIO INC., Tokyo, Japan) was 0.077 (Aβ42 392 pg/mL, Aβ40 5117 pg/mL), which was above the cut-off value of 0.067. CSF p-tau181 (26.7 pg/mL) and t-tau (202 pg/mL) measured by ELISA were both within the normal range. The patient was unable to eat and did not want alternative nutrition such as tube feeding, and died one month later. The autopsy was performed at a postmortem interval of 17 h 40 min. Macroscopic image of the brain. The brain weighed 974 g. Convexity atrophy was observed in the bilateral frontal and left parietal lobes (A). Anti-Aβ immunostaining (mouse monoclonal, clone 12B2; IBL, Japan; 1:50) showed only few diffuse plaques limited to the neocortex and transentorhinal cortex (B: parietal cortex, C: entorhynal cortex) with no neuritic plaques (Thal phase 2, CERAD none). Amyloid angiopathy was confined to few small vessel walls in the neocortex, cerebellum, and hippocampus (D). Gallyas stain showed flame-shaped neurofibrillary tangles limited to the entorhynal gyrus and transentorhynal gyrus (Braak NFT stage (rt./lt.) (Ⅱ/Ⅱ) (E). Astrocytic plaques were observed with anti phosphorylated tau immunostaining (mouse monoclonal, clone AT8; Innogenetics, Ghent, Belgium; 1:1000) (F) and Gallyas stain (G). Ballooned neuron was observed which were positive for 4-repeat tau (mouse monoclonal, clone RD4; Upstate, Lake Placid, NY, USA 1:200) (H) but not for 3-repeat tau (mouse monoclonal, clone RD3; Upstate, Lake Placid, NY, USA 1:8000) (I). These findings were consistent with the neuropathological diagnosis of corticobasal degeneration (CBD).
Scale bars: 2cm: (A),100µm: (B, C), 250µm (25µm: inset): (D, E), 25µm: (F, G), 20µm: (H, I)
